# A case of neurosarcoidosis presenting as hippocampal sclerosis: Clinicopathological correlation and proposed mechanistic link

**DOI:** 10.1016/j.ebr.2025.100837

**Published:** 2025-11-07

**Authors:** Elham Rahimian, Guive Sharifi, Hans Jürgen Huppertz, Majid Reza Tahsini, Behnam Safarpour Lima, Elham Naderi, Yalda Nilipour, Melika Javani, Soudeh Ghafouri-Fard

**Affiliations:** a Division of Neuroradiology, Haghighat Medical Imaging Research Center, Haghighat Medical Imaging Center, Tehran, Iran; b Skull Base Research Center, Loghman Hakim Hospital, Shahid Beheshti University of Medical Sciences, Tehran, Iran; c Swiss Epilepsy Center, Zurich, Switzerland; d Department of Neurology, School of Medicine, Shahid Beheshti University of Medical Sciences, Tehran, Iran; e Pediatric Pathology Research Center, Research Institute for Children’s Health, Shahid Beheshti University of Medical Sciences, Tehran, Iran; f Neuromuscular Research Center, Tehran University of Medical Sciences, Tehran, Iran; g Shahid Beheshti University of Medical Sciences, Tehran, Iran

**Keywords:** Neurosarcoidosis, Hippocampal sclerosis, Mesial temporal lobe epilepsy, Inflammatory epilepsy, Granulomatous inflammation

## Abstract

Neurosarcoidosis is a rare manifestation of systemic sarcoidosis, typically affecting the meninges, cranial nerves, and hypothalamic–pituitary axis. Parenchymal brain involvement—particularly of the hippocampus—is uncommon and only rarely associated with epilepsy or hippocampal sclerosis (HS). Here, we describe a rare coexistence of biopsy-proven neurosarcoidosis and hippocampal sclerosis, suggesting secondary hippocampal injury due to chronic inflammation presenting with drug-resistant right mesial temporal lobe epilepsy (MTLE). We reviewed the clinical electrophysiological imaging and histopathological findings of a 47-year-old male who underwent anterior temporal lobectomy (ATL) after seizure onset which was localized to the right anterior to mid temporal region. Preoperative MRI was consistent with mesial temporal sclerosis. Histopathological analysis revealed both classic features of HS (neuronal loss and gliosis) and non-caseating granulomas diagnostic of neurosarcoidosis distributed within the medial temporal lobe and involving the amygdala. These findings suggest chronic inflammation may have contributed to hippocampal injury and epileptogenesis. The patient became seizure-free postoperatively. This case underscores the potential for neurosarcoidosis to mimic isolated MTLE and coexist with HS as a rare co-localized pathology. It raises the hypothesis that immune-mediated inflammation may play a pathogenic role in hippocampal degeneration. Recognition of such cases may enhance diagnostic accuracy and inform surgical planning in epilepsy associated with systemic inflammatory disorders.

## Highlights

• Neurosarcoidosis may present without systemic symptoms.
• Chronic immune-mediated inflammation may contribute to hippocampal degeneration.
• MRI may not reveal hippocampal involvement in neurosarcoidosis.

## 1 Introduction

Sarcoidosis is a chronic granulomatous disorder of unknown etiology that most commonly affects the lungs and lymphatic system but may involve virtually any organ, including the central nervous system (CNS) [1]. Neurosarcoidosis occurs in approximately 5–15 % of patients with systemic sarcoidosis and typically manifests as cranial neuropathies, meningeal inflammation, or hypothalamic–pituitary axis dysfunction. Parenchymal brain involvement is less frequent and rarely presents with focal epilepsy [2].

We describe a rare case of biopsy-confirmed neurosarcoidosis in a patient with drug-resistant right temporal lobe epilepsy who exhibited classic imaging findings of hippocampal sclerosis. Histopathology of resected brain tissue revealed both non-caseating granulomas and hallmark features of HS, confirming coexisting neurosarcoidosis and hippocampal sclerosis, consistent with secondary inflammatory injury rather than independent dual pathology. This case highlights the importance of considering neurosarcoidosis in the differential diagnosis of mesial temporal lobe epilepsy (MTLE) and supports the effectiveness of epilepsy surgery—even in the context of systemic inflammatory disease.

## 2 Case presentation

A 47-year-old right-handed man with a history of hypertension, hyperlipidemia, and biopsy-confirmed systemic sarcoidosis presented with a six-year history of focal seizures. His initial episodes began with an olfactory aura described as a vinegar-like smell, followed by hypersalivation and lightheadedness. These focal seizures with preserved consciousness were brief (<30 s) and occurred one to two times per month. Approximately one year after onset, he experienced three generalized tonic–clonic seizures. He was started on valproate, later supplemented by levetiracetam, which eliminated generalized seizures but failed to control focal seizures with impaired consciousness, now presenting as brief oral automatisms.

**Fig. 1:**
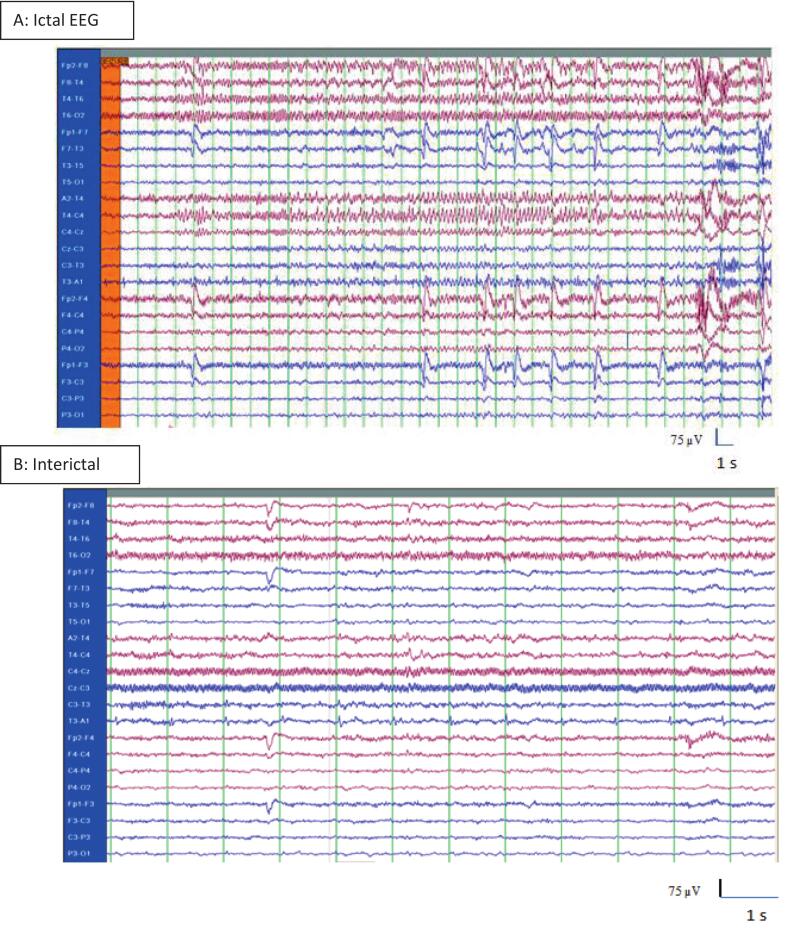
(A) Ictal EEG showing right anterior to mid-temporal rhythmic alpha activity evolving into lower-frequency, higher-amplitude waves, consistent with seizure evolution. The time base was reduced from 30 mm/s to 10 mm/s to display the entire ictal episode within a single epoch. The patient remained aware but immobile and reported experiencing olfactory hallucinations during the seizure (time base = 10 mm/s; sensitivity = 7.5 µV/mm). (B) Interictal EEG showing right anterior to mid-temporal focal sharp wave (time base = 30 mm/s; sensitivity = 7.5 µV/mm).

The ictal EEG showed rhythmic alpha activity over the right anterior to mid-temporal region, evolving into lower-frequency, higher-amplitude discharges, consistent with seizure evolution. Interictally, the EEG demonstrated focal sharp waves in the same region, supporting a right temporal seizure focus. Despite medication adherence, seizures persisted, prompting referral for surgical evaluation (Fig. 1).

**Fig. 2:**
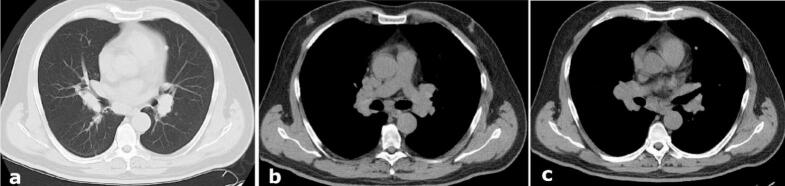
(a–c) Chest Computed tomography (CT) images showing bilateral hilar lymphadenopathy and interstitial nodular opacities suggestive of pulmonary sarcoidosis.

The patient's sarcoidosis had been diagnosed two years before seizure onset after presenting with sternal lymphadenopathy. Mediastinal CT showed bilateral hilar and parenchymal lymphadenopathy; biopsy revealed non-caseating granulomas. The patient had not received corticosteroids or immunosuppressive therapy prior to epilepsy onset. A transient episode of pulmonary atelectasis occurred following biopsy (Fig. 2).

Presurgical workup included 72-hour video EEG monitoring, which showed interictal right anterior to mid temporal slowing and spikes. One seizure with preserved consciousness was captured, confirming a right anterior to mid-temporal seizure onset zone. Neurological examination was normal, and no risk factors such as trauma, CNS infections, or febrile seizures were reported.

**Fig. 3:**
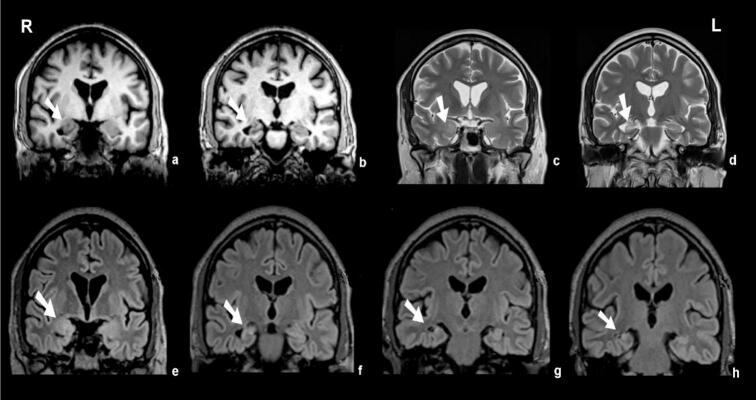
Coronal preoperative MR images demonstrating right mesial temporal abnormalities. T1-weighted images (a, b), and T2-weighted images (c, d), FLAIR (fluid-attenuated inversion recovery) images (e, f, g, h), obtained through the amygdala and the head of the hippocampus show right hippocampal atrophy with T2/FLAIR hyperintensity consistent with hippocampal sclerosis, accompanied by patchy signal changes in the right amygdala (arrows). R = Right L = Left.

MRI revealed classical features of right hippocampal sclerosis, including volume loss, T2W/FLAIR hyperintensity, T1W hypointensity, and less defined hippocampal lamination. Considerable findings included a signal change in the right amygdala, while the left hippocampus was normal. No other structural abnormalities were identified (Fig. 3). The patient underwent right anterior temporal lobectomy with amygdalohippocampectomy. Postoperatively, he developed transient respiratory distress due to pulmonary embolism, which was managed without complications. He was discharged without new neurological deficits.

## 3 Histopathology and Follow-Up

**Fig. 4:**
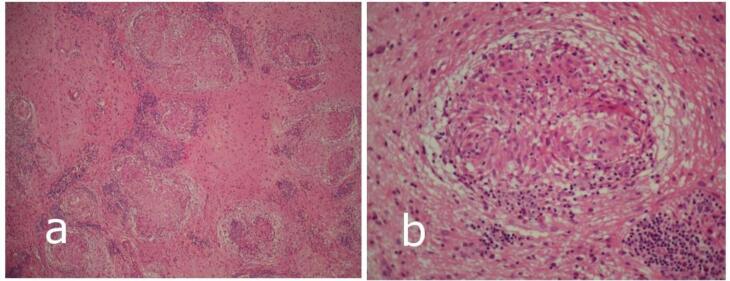
Histopathological sections from the medial temporal lobe, including the amygdala, demonstrating non-caseating granulomatous inflammation consistent with neurosarcoidosis. (a) Low power view showing multiple well-formed granulomas composed of epithelioid histiocytes and occasional multinucleated giant cells surrounded by lymphocytic cuffs within the neuropil. (Hematoxylin and eosin × 100). (b) High power view of a small granuloma adjacent to a blood vessel with prominent perivascular lymphocytic infiltration (Hematoxylin and eosin × 400).

Histopathological analysis demonstrated two key findings: (1) hippocampal neuronal loss with associated astrogliosis consistent with probable hippocampal sclerosis according to the ILAE classification (Blümcke et al., 2013), the designation “probable” reflecting the fragmented nature of the resected tissue, which precluded complete subfield assessment; and (2) non-caseating granulomatous inflammation consistent with neurosarcoidosis. Multiple small granulomas accompanied by perivascular lymphocytic infiltration were distributed throughout the medial temporal lobe and involved the amygdala (Fig. 4, Fig. 4).

There were no granulomas in the hippocampus or at the resection margins of the lobectomy, but they were clearly present in the proximal medial temporal structures, including the amygdala.

Postoperative MRI showed cystic encephalomalacia in the resection cavity without enhancement or residual disease. At one-year and subsequent 18-month follow-up visits, the patient remained completely seizure-free and neurologically intact. He continued on valproate and levetiracetam and was started on methotrexate and low-dose prednisolone for systemic sarcoidosis.

## 4 Discussion

Mesial temporal lobe epilepsy (MTLE) associated with hippocampal sclerosis (HS) is typically characterized by hippocampal atrophy with T2/FLAIR hyperintensity and confirmed by concordant electroclinical findings [3]. Our patient fulfilled these radiological and electroclinical criteria, leading to a preoperative diagnosis of HS. Histopathological analysis, however, revealed an additional and unexpected finding—non-caseating granulomatous inflammation involving the medial temporal lobe and amygdala—suggesting an inflammatory contribution to hippocampal injury.

Microscopically, two key findings were identified. The hippocampus showed neuronal loss and astrogliosis consistent with HS; however, because the resected tissue was fragmented, classification according to the ILAE system was limited to probable HS. In this context, the designation of probable HS reflects a technical limitation rather than diagnostic uncertainty. Although subfield evaluation could not be completed, the preoperative MRI and concordant EEG findings fulfill the criteria for definite HS in a multimodal diagnostic context. The MRI demonstrated the classical features of HS—global hippocampal volume loss with T2/FLAIR hyperintensity, T1 hypointensity, and reduced internal definition—findings consistent with definite HS from a radiological perspective.

Adjacent medial temporal structures, including the amygdala, contained well-formed non-caseating granulomas with perivascular lymphocytic infiltration, diagnostic of neurosarcoidosis. The coexistence and close proximity of these lesions support the hypothesis that a chronic, subclinical inflammatory process contributed to hippocampal injury rather than being coincidental. Notably, no radiological signs of sarcoidosis were evident preoperatively, underscoring that such inflammation can remain clinically and radiographically silent.

While neurosarcoidosis is a recognized but uncommon manifestation of systemic sarcoidosis, parenchymal involvement typically affects the brainstem, basal ganglia, and periventricular white matter [2]. Hippocampal or amygdalar disease is exceptionally rare. Christoforidis et al. [4], Shah et al. [5], and Padilha et al. [6] described extensive CNS and meningeal manifestations without hippocampal lesions, emphasizing leptomeningeal and hypothalamic–pituitary involvement as the predominant imaging findings. In this context, the detection of granulomatous inflammation within the amygdala and medial temporal lobe in our case represents an unusual and underrecognized pattern of neurosarcoid involvement with potential epileptogenic relevance. Previous reports have described mass-like parenchymal lesions mimicking neoplasms—such as those by Sari et al. [7], Elia et al. [8], Hodge et al. [9], and Sponsler et al. [10]—yet none demonstrated concurrent hippocampal sclerosis or proposed chronic inflammation as a mechanism for hippocampal degeneration. Hippocampal involvement remains notably absent from large series and imaging reviews [[4], [5], [6],^11^]. To our knowledge, this is the first histologically confirmed case of biopsy-proven neurosarcoidosis coexisting with hippocampal sclerosis. This highlights a rare inflammatory etiology of HS within the same temporal lobe and suggests that sustained granulomatous inflammation may contribute to epileptogenesis in selected patients.

A small number of reports have explored the association between neurosarcoidosis and epilepsy, but none have documented the co-localized pathology confirmed in our case. Malmgren et al. [12] described a patient with neurosarcoidosis and pharmacoresistant right temporal lobe epilepsy who achieved seizure reduction after anterior temporal resection, yet without classical HS. Mandyam et al. [13] reported steroid-responsive seizures in systemic sarcoidosis without histological confirmation. In contrast, our case provides direct microscopic evidence of both well-formed granulomas and hippocampal sclerosis within the same temporal lobe, reinforcing the hypothesis that chronic immune-mediated inflammation can precipitate hippocampal injury and secondary sclerosis.

**Fig. 5:**
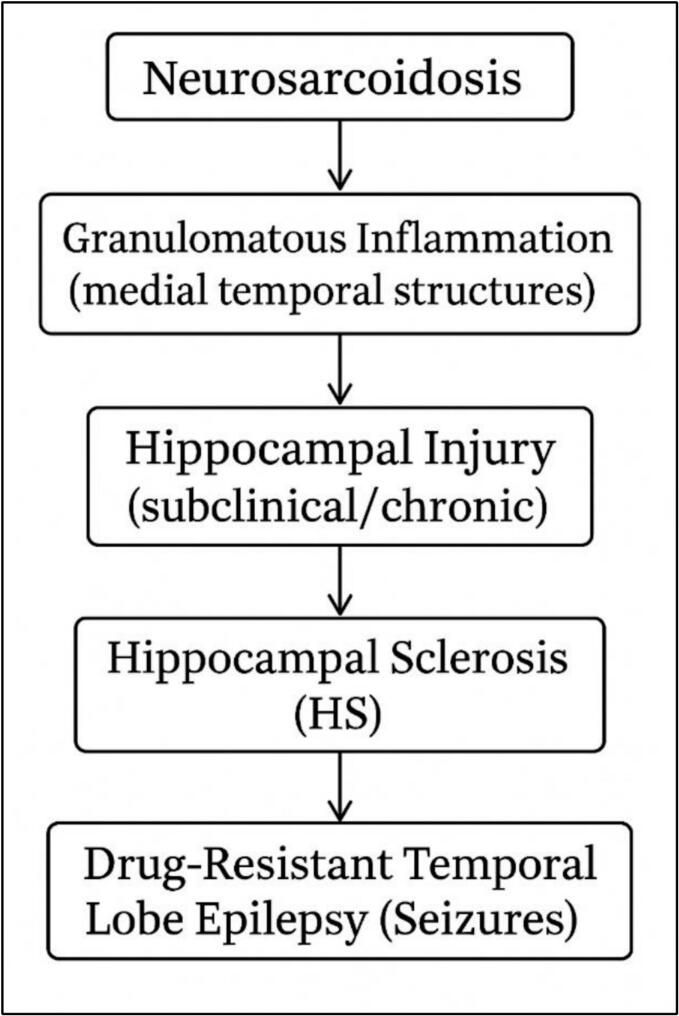
Proposed causal relationship between granulomatous inflammation in neurosarcoidosis and hippocampal sclerosis.

The coexistence of granulomatous inflammation and hippocampal neuronal loss within the same resected tissue strongly supports a causal rather than incidental link between chronic inflammation and hippocampal sclerosis. The hippocampus is highly vulnerable to inflammatory, ischemic, and excitotoxic injury. Persistent granulomatous inflammation may disrupt hippocampal microarchitecture and impair regional perfusion through sarcoid-related vasculitis, thereby promoting progressive neuronal loss (Fig. 5).

In MTLE, pro-inflammatory signaling (e.g., IL-1β/TNF-α from activated microglia and astrocytes) lowers seizure threshold, drives excitotoxic injury, and sustains gliosis, changes that mirror MRI atrophy and T2/FLAIR hyperintensity in HS [14,15]. Sarcoid-related perivascular granulomatous inflammation may add vascular and microstructural stressors (vasculitic hypoperfusion, blood–brain barrier dysfunction), amplifying injury within the hippocampal circuitry [14,15]. Collectively, these findings support the hypothesis that chronic granulomatous inflammation, as seen in neurosarcoidosis involving the temporal lobe, may initiate or accelerate hippocampal sclerosis through convergent immune-mediated mechanisms. Clinically, the patient’s seizure semiology, EEG findings, and postoperative course were entirely consistent with right MTLE [3]. Complete seizure freedom following anterior temporal lobectomy confirms that, despite the presence of inflammation, the hippocampus remained the principal epileptogenic focus. Similar favorable outcomes have been reported in selected cases of sarcoidosis-related epilepsy, where surgical resection achieved seizure control despite underlying inflammatory pathology [12,13]. This observation emphasizes that systemic inflammatory disease is not a contraindication to epilepsy surgery when anatomical and electroclinical localization are concordant. It also underscores the importance of histopathological evaluation even when imaging and clinical data suggest a classical presentation of HS, as unexpected inflammatory or immune-mediated mechanisms may coexist and influence epileptogenesis [16].

## 5 Conclusion

In summary, this case broadens the differential diagnosis of MTLE and highlights the diagnostic and pathophysiological complexity of neurosarcoidosis presenting as medically intractable mesial temporal lobe epilepsy. This is the first report of histologically confirmed hippocampal sclerosis coexisting with non-caseating granulomas in the same temporal lobe, raising the possibility that chronic immune-mediated inflammation may contribute to the development of hippocampal damage and epileptogenesis. Recognition of this potential relationship may expand our understanding of atypical HS etiologies and support the consideration of systemic inflammatory conditions in differential diagnoses. Furthermore, this case demonstrates that standard anterior temporal lobectomy can be curative even in the context of inflammatory pathology, provided that seizure localization is clear and concordant.

## Data availability

Not applicable.

## CRediT authorship contribution statement

**Elham Rahimian:** Data curation, Conceptualization. **Guive Sharifi:** Investigation, Data curation. **Hans Jürgen Huppertz:** Investigation, Data curation. **Majid Reza Tahsini:** Investigation, Formal analysis. **Behnam Safarpour Lima:** Data curation. **Elham Naderi:** Investigation, Data curation. **Yalda Nilipour:** Data curation. **Melika Javani:** Data curation. **Soudeh Ghafouri Fard:** Investigation.

## Funding

Not applicable.

**Compliance with Ethical Standards**

Related protocols were approved by Haghighat medical imaging research center, Haghighat medical imaging center, Tehran, Iran. Informed consent forms were signed by their parents. All methods were performed in accordance with relevant guidelines and regulations.

**Consent to participate**

Informed consent forms were signed by guardians of patients.

**Consent to Publish**

Informed consent forms were signed by guardians of patients.

**Declaration of generative AI use**

During the preparation of this work the author(s) did not used any AI-assisted technologies.

## Declaration of competing interest

The authors declare that they have no known competing financial interests or personal relationships that could have appeared to influence the work reported in this paper.
